# Lessons Learned From Methodological Validation Research in E-Epidemiology

**DOI:** 10.2196/publichealth.5880

**Published:** 2016-10-18

**Authors:** Emmanuelle Kesse-Guyot, Karen Assmann, Valentina Andreeva, Katia Castetbon, Caroline Méjean, Mathilde Touvier, Benoît Salanave, Valérie Deschamps, Sandrine Péneau, Léopold Fezeu, Chantal Julia, Benjamin Allès, Pilar Galan, Serge Hercberg

**Affiliations:** ^1^ Équipe de Recherche en Epidémiologie Nutritionnelle (EREN), Centre de Recherche en Epidémiologie et Statistiques, COMUE Sorbonne Paris Cité, Inserm (U1153), Inra (U1125), Cnam Université Paris 13 Bobigny France; ^2^ Ecole de Santé Publique, Centre de Recherche en Epidémiologie, Biostatistiques et Recherche Clinique Université Libre de Bruxelles Bruxelles Belgium; ^3^ Unité de Surveillance et d’Epidémiologie Nutritionnelle (USEN), Institut de Veille Sanitaire, Centre de Recherche en Epidémiologie et Statistiques, COMUE Sorbonne Paris Cité Université Paris 13 Bobigny France; ^4^ Hôpital Avicenne Département de Santé Publique Bobigny France

**Keywords:** cohort studies, bias, epidemiology

## Abstract

**Background:**

Traditional epidemiological research methods exhibit limitations leading to high logistics, human, and financial burden. The continued development of innovative digital tools has the potential to overcome many of the existing methodological issues. Nonetheless, Web-based studies remain relatively uncommon, partly due to persistent concerns about validity and generalizability.

**Objective:**

The objective of this viewpoint is to summarize findings from methodological studies carried out in the NutriNet-Santé study, a French Web-based cohort study.

**Methods:**

On the basis of the previous findings from the NutriNet-Santé e-cohort (>150,000 participants are currently included), we synthesized e-epidemiological knowledge on sample representativeness, advantageous recruitment strategies, and data quality.

**Results:**

Overall, the reported findings support the usefulness of Web-based studies in overcoming common methodological deficiencies in epidemiological research, in particular with regard to data quality (eg, the concordance for body mass index [BMI] classification was 93%), reduced social desirability bias, and access to a wide range of participant profiles, including the hard-to-reach subgroups such as young (12.30% [15,118/122,912], <25 years) and old people (6.60% [8112/122,912], ≥65 years), unemployed or homemaker (12.60% [15,487/122,912]), and low educated (38.50% [47,312/122,912]) people. However, some selection bias remained (78.00% (95,871/122,912) of the participants were women, and 61.50% (75,590/122,912) had postsecondary education), which is an inherent aspect of cohort study inclusion; other specific types of bias may also have occurred.

**Conclusions:**

Given the rapidly growing access to the Internet across social strata, the recruitment of participants with diverse socioeconomic profiles and health risk exposures was highly feasible. Continued efforts concerning the identification of specific biases in e-cohorts and the collection of comprehensive and valid data are still needed. This summary of methodological findings from the NutriNet-Santé cohort may help researchers in the development of the next generation of high-quality Web-based epidemiological studies.

## Introduction

Advances in knowledge on health and disease strongly rely on the availability, quality, and comprehensiveness of data from prospective cohort studies with very large population-based samples. Such studies are particularly important for the exploration of research hypotheses that do not lend themselves to randomized trials.

Traditional methods of data collection in large epidemiological studies (paper-and-pencil questionnaires, face-to-face interviews, and telephonic interviews) require immense resources in terms of logistics (mailing, preparation and postage, transportation, etc); personnel (interview staff, administrative help, data entry, and data quality control); and resources (printing paper, heavy-duty printers, toner cartridges, etc). In addition, traditional methods likely make study participation burdensome (eg, mailing back questionnaires, organizing appointments, transportation time to get to examination centers). As traditional epidemiological studies have been witnessing a steady decline in response rates over the last few decades [1] that has resulted in concerns about selection bias and representativeness, alternative strategies for the recruitment and follow-up of participants are gaining momentum. Although self-selection bias does not systematically lead to erroneous conclusions in etiological research, it has major implications for investigations focusing on social determinants and for descriptive and prevalence studies [2]. Furthermore, limitations of traditional methods include interviewer-related biases and a high susceptibility to errors during data entry.

Although both traditional and e-epidemiological research relies on volunteers, the use of innovative, computerized tools could provide highly pertinent future directions [3-7]—especially in France, where 82% of the population has an access to the Internet [8].

Currently, most existing e-cohorts include young women as target populations, and are characterized by a high proportion of well-educated volunteers [9-14]. Thus far, few studies have evaluated e-cohorts with respect to participant selection processes or sociodemographic profiles, or have validated tools for Web-based epidemiological research [15-17].

In this context, the aim of this work was to compile methodological research findings from validation studies based on the ongoing NutriNet-Santé e-cohort with >100,000 participants enrolled till date.

## Methods

### General Description of the NutriNet-Santé Study

The NutriNet-Santé study [18] is a Web-based cohort launched in France in 2009, with a planned 10-year follow-up. Its main objective was the comprehensive investigation of the relationship between multiple aspects of nutrition and health [18]. Participants were recruited through a combination of Web-based and traditional recruitment strategies such as television and radio broadcasts, newspaper advertising, and flyer distribution. A secured website was used as a platform for study inclusion, data collection, and follow-up [19]. Inclusion criteria were residence in France, age ≥18 years, and access to the Internet. Participants were followed via the Internet and were asked to complete Web-based questionnaires on a regular basis. The study was approved by the ethics committee of the French Institute for Health and Medical Research (IRB INSERM n° 0000388 FWA00005831) and by the National Commission on Informatics and Liberty (CNIL n°908450 and n° 909216). All subjects signed an electronic informed consent. All data collection instruments were Web-based and provide the participants with general instructions as well as automated alerts (text, pictures, and error messages) designed to improve the accuracy and completeness of the responses.

### Sociodemographic Data

Sociodemographic data (age, gender, education, marital status, number of children, geographical region, and occupational status) were collected via Web-based questionnaires.

Participants were also asked to complete questionnaires inquiring about motives for participation (“Would you have participated to the NutriNet-Santé study if it was not Internet-based?” [yes or no]), computer and Internet skills, and channels of recruitment. They were also asked to provide part of their 15-digit national identification number (personal Social Security number).

A study was conducted to compare the self-administered Web-based version of the sociodemographic questionnaire with a traditional paper version among 147 participants (paper first, n=76; Web-based first, n=71).

### Dietary Data

Dietary data were collected at inclusion and on a biannual basis via a set of 3 24-h records (24 h) and randomly allocated over a 2-week period, including 2 weekdays and 1 weekend day. Participants reported all foods and beverages (type and quantity) consumed at each meal (breakfast, lunch, and dinner) or any other eating occasion during a 24-h period. Each food or beverage item consumed was entered into the system via a food browser or a search engine. Additional information about time and place of eating were also collected. Portion sizes were estimated on the basis of the estimated weight of a food item, purchase units or of photographs from a validated picture booklet [20], reflecting more than 250 generic foods corresponding to more than 2000 specific food items, presented in 7 different portion sizes. Nutrient intakes were calculated using the ad hoc NutriNet-Santé composition table including more than 2000 foods and beverages [21]. In addition to the 24-h dietary records, a previously validated semiquantitative food-frequency questionnaire was proposed to the NutriNet-Santé participants 6 months after study inclusion [22].

**Figure 1:**
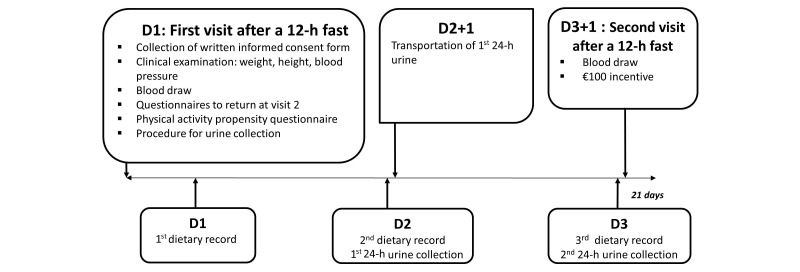
Design of the dietary data validation study, NutriNet-Santé, 2013 (N=199).

A validation study was conducted to evaluate the quality of the data collected via the 24-h dietary record tool against 24-h urinary and plasma biomarkers [23,24]. A total of 199 adult volunteers were included, who completed 3 24-h dietary records and provided 2 24-h urine samples and 2 blood samples concomitant with the first and third 24-h dietary record. Figure 1 illustrates the design of this validation study in a detailed manner.

Beyond the objective validity of the 24-h tool, we also carried out a comparative study to evaluate the concordance of results obtained by the 24-h Web tool with results obtained via telephonic interviews conducted by a dietician (which is the reference method used when a traditional mode of survey is administered) [25]. This study also included a comparison of the estimated financial cost related to the implementation and use of the Web-based 24-h dietary records with the cost related to the standard assessment involving dieticians.

### Anthropometric Data

Anthropometric data including weight, height, restrictive dieting, and weight history were collected through a self-administered questionnaire at inclusion and annually thereafter. In addition, a subsample of participants (n=20,000) completed clinical evaluations and biological sampling during clinic visits held throughout France. During these visits, participants underwent a clinical examination including weight, height, waist circumference, hip circumference, and bioimpedance measurements. In order to evaluate the quality of the self-reported anthropometric data, a random subsample of all participants with a scheduled clinical examination were asked to complete a Web-based anthropometric questionnaire 3 days before their appointment (November 2011-July 2012) [26]. Among this subsample of participants, a further subsample was randomly selected and asked to verbally report their height and weight to a trained technician on the day of the examination before being measured.

An additional comparative study was set-up in order to compare the Web-based anthropometric questionnaire with a paper-based version of the same questionnaire [27].

### Statistical Analyses Performed in the Different Validation Studies

In order to provide a detailed presentation of the sociodemographic profiles of the study population, characteristics of 122,912 NutriNet-Santé participants (recruited between 2009 and 2014) were compared with data from the French 2009 Census [28]. Moreover, the impact of a statistical weighting method developed to enhance the representativeness of descriptive or analytical results was evaluated, using the SAS macro “CALMAR (CALage sur MARges)” provided by the Institut national de la statistique et des études économiques (INSEE) [29]. This macro was developed to recover a population-representative sample from nonrepresentative samples obtained by surveys by weighting individual data using ancillary information. In the study on demographic data in the NutriNet-Santé cohort, weights were calculated based on gender, age, birthplace, educational level, occupation, marital status, presence of children in the household, and area of residence.

Dietary data in the NutriNet-Santé cohort were compared with nationally representative data from the cross-sectional Etude Nationale Nutrition Santé (ENNS, 2006-2007), including 2754 French adults aged 18-74 years [30].

In both studies, reported dietary intakes from 3 (nonconsecutive days over a 2-week period, including 2 weekdays and 1 weekend day) 24-h dietary surveys (computerized records in the NutriNet-Santé study and telephone recalls in the ENNS) were weighted using the French Census estimates. The same food composition table was used to estimate nutrient intakes in both samples.

Several statistical indicators were used to analyze the data including means, standard errors of the mean, intraclass correlation coefficients (ICCs), kappa coefficients, and Spearman correlation coefficients. Self-reported dietary intakes of protein, potassium, and sodium were compared with intakes estimated on the basis of urinary biomarkers, using a validated log-ratio formula [23]. Both simple Spearman correlation coefficients and coefficients adjusted for age, BMI, smoking, education, energy intake, alcohol consumption, and use of dietary supplements were calculated.

## Results

### Summary

Our findings are based on 12 studies investigating characteristics and representativeness of the sample [28], comparison of dietary data with national findings [31], recruitment [32], motives for participation [33], Internet or computer skills [34], and quality of the data [23-27,35,36].

### Characteristics and Representativeness

**Figure 2:**
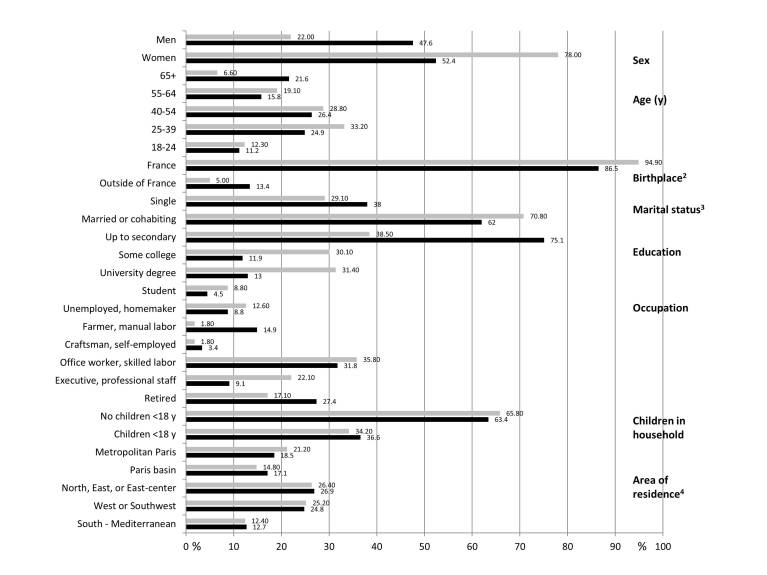
Comparison of the sociodemographic characteristics of NutriNet-Santé (2009-2014) subjects (N=122,912) with French census data. Gray bars denote NutriNet-Santé subjects. Black bars denote French Census estimates (2009) for individuals aged 18 years and above in metropolitan France from INSEE. All differences between NutriNet-Santé subjects and the Census estimates were statistically significant (all chi-square–test *P* values were <.001).^2^France: including Corsica and overseas territories.^3^Single: never-married, widowed, divorced, or separated.^4^Geographical distribution based on the Zone d'études et d'aménagement du territoire (ZEAT) defined by INSEE. INSEE: Institut national de la statistique et des études économiques.

The gender ratio (females:males) in the e-cohort was 5:3 and the average age of participants at inclusion was 42.6 (SD 14.6) years. A small proportion, that is, 5.00% (6208/122,912) of participants were born outside of France, 31.40% (38,606/ 122,912) had university-level education, 70.80% (87,048/ 122,912) were married or cohabiting, and 65.80% (80,934/ 122,912) did not have children aged under 18 years in their household. Figure 2 presents the crude (unweighted) sociodemographic characteristics of the NutriNet-Santé study population in comparison with the French Census data from 2009 [28]. The NutriNet-Santé sample was relatively similar to the national data concerning the geographical area of residence and the presence of children in the household.

Similarly, the age distribution up to 65 years resembled the respective data observed in the general French population. However, important discrepancies were observed concerning gender, education, and occupation. Some subgroups are highly underrepresented in the e-cohort (eg, farmers), limiting the effectiveness of the weighting method and thus the generalizability. It is noteworthy that the proportion of volunteers who were unemployed or homemakers or disabled was higher in the NutriNet-Santé sample than in the general French population.

### Comparison of Dietary Data With National Findings

**Figure 3:**
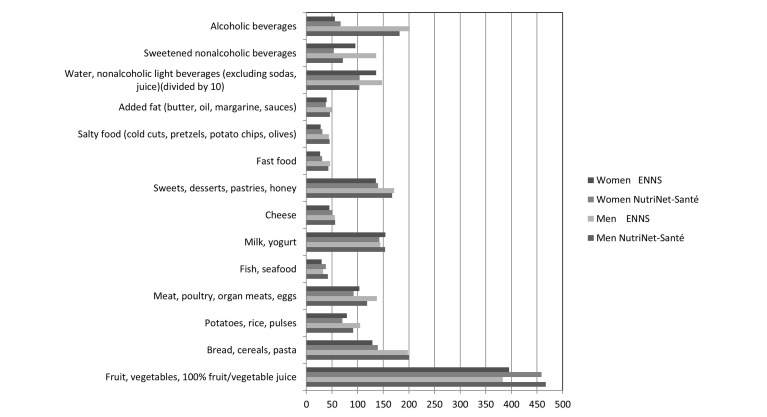
Mean food (g/d) and beverage (ml/d) intake in the NutriNet-Santé study (2009-2010, N=49,443) and the nationally representative survey (ENNS, 2006-2007, n=2754). All data from both NutriNet-Santé and ENNS are weighted for age, education, presence of children in household, and season of data collection, using French 2007 Census data. ENNS: Etude Nationale Nutrition Santé.

A comparison of dietary intake in the NutriNet-Santé and the nationally representative ENNS is shown in Figure 3 and Table 1 [31].

The comparison revealed that the consumption of fruits and vegetables and fish and seafood was higher in the NutriNet-Santé cohort. In contrast, the consumption of potatoes and pulses, meat and eggs, and nonalcoholic beverages was significantly lower in the NutriNet-Santé cohort than in the ENNS.

**Table 1:** Intake of nutrients in the NutriNet-Santé study (2009-2010, N=49,443) and the nationally representative survey (ENNS, 2006-2007, n=2754)^a,b^.

Nutritional indicators	Men		Women	
	NutriNet-Santé	ENNS^c^	NutriNet-Santé	ENNS
Total energy (Kcal/d)	2326.31 (5.79)	2388.67 (27.70)	1767.94 (2.21)	1713.69 (14.02)
Total carbohydrates (g/d)	238.61 (0.69)	246.11 (3.33)	184.04 (0.27)	180.35 (1.69)
Total lipids (g/d)	97.11 (0.32)	98.44 (1.27)	75.95 (0.13)	73.30 (0.74)
Protein (g/d)	96.13 (0.26)	98.31 (1.13)	75.72 (0.10)	74.10 (0.70)
Dietary fiber (g/d)	21.30 (0.08)	19.12 (0.30)	18.17 (0.03)	16.10 (0.19)
Calcium (mg/d)	1028.98 (3.48)	1022.16 (13.91)	879.87 (1.50)	869.80 (9.94)
Retinol (μg/d)	608.97 (8.01)	668.23 (37.71)	480.38 (3.72)	496.11 (27.02)
Beta-carotene (μg/d)	3418.12 (27.22)	3196.52 (113.87)	3270.61 (13.40)	3211.01 (67.38)
Vitamin B6 (mg/d)	2.04 (0.01)	1.89 (0.02)	1.63 (0.00)	1.52 (0.02)
Vitamin B9 (μg/d)	352.74 (1.27)	332.61 (4.78)	312.18 (0.59)	292.16 (3.15)
Vitamin B12 (μg/d)	6.36 (0.06)	6.17 (0.21)	5.02 (0.03)	4.66 (0.14)
Vitamin C (mg/d)	117.53 (0.78)	95.87 (3.49)	109.87 (0.44)	96.68 (1.70)
Vitamin D (μg/d)	2.93 (0.02)	2.47 (0.10)	2.50 (0.01)	2.00 (0.06)
Vitamin E (mg/d)	13.16 (0.05)	11.72 (0.22)	11.10 (0.03)	9.62 (0.12)
Zinc (mg/d)	13.06 (0.04)	13.09 (0.18)	10.29 (0.02)	9.67 (0.11)
Iron (mg/d)	15.63 (0.06)	14.00 (0.20)	12.45 (0.02)	10.80 (0.12)
Potassium (mg/d)	3344.58 (9.15)	3194.87 (38.86)	2840.51 (3.99)	2668.44 (24.74)
Magnesium (mg/d)	372.75 (1.19)	329.68 (3.68)	310.03 (0.52)	266.18 (2.54)

^a^All data from both NutriNet-Santé and ENNS are weighted for age, education, presence of children in the household, and season of data collection, using French 2007 Census figures.

^b^Values are means with SEs of the means within the brackets.

^c^ENNS: Etude Nationale Nutrition Santé.

Total energy and macronutrient intake was similar overall, whereas intake of fiber, iron, magnesium, and vitamins B6, B9, C, D, and E were higher in the NutriNet-Santé study than in the ENNS. Overall, true differences in intake, differential measurement errors related to the different modes of administration, sample composition (volunteers vs stratified, random sample), and volunteer bias might each contribute to explaining the findings. Concerning potential volunteer bias in the NutriNet-Santé study, it should be noted the magnitude of such bias should be similar in volunteer-based nutritional epidemiologic studies that use traditional methods of data collection. Given the large samples in both the NutriNet-Santé study and the ENNS study, it is noteworthy that not all statistically significant differences are practically meaningful.

### Recruitment, Motives for Participation, and Internet or Computer Skills

Efforts for diversifying communication channels for sample recruitment could be an important element for increasing participant profile heterogeneity. We have thus investigated the different channels through which subjects were ultimately recruited according to sociodemographic profiles [32].

The largest number of subjects was reached using television advertisements (34.46%, 30,414/88,238). Other channels with substantial importance were radio broadcasts (16.21%, 14,309 out of 88,238) and websites (19.05%, 16,807/88,238). Note that compared with other channels, among subjects recruited by television broadcasts, there was a higher proportion of individuals with a low educational level (44.1% vs 37.4% in the whole sample) and of unemployed individuals (36.1% vs 4.8% in the whole sample).

As specific profiles of subjects involved in nutritional Web-based cohorts may be related to specific motives for participation, we have investigated reasons and motives for participation in the NutriNet-Santé study according to sociodemographic characteristics [33]. The use of Internet as an exclusive means of follow-up was an important source of motivation for participation, especially among men, young individuals, those of high socioeconomic status, and among obese persons.

A substantial portion (23.79%, 10,235 out of 43,028) of subjects in the NutriNet-Santé study has relatively low self-reported computer or Internet skills, suggesting that this factor does not represent a barrier to participation in Web-based cohorts [34]. Besides, several subgroups of subjects with lower computer skills (eg, women or those with lower educational level) seemed more inclined to accept the demands associated with participation in the Web cohort [34].

### Quality of the Collected Data

#### Sociodemographic Data

Agreement between data obtained from the self-administered Web-based version and the traditional paper version of the sociodemographic questionnaire was high, with ICCs for continuous variables ranging between .81 and 1 and kappa coefficients for categorical variables ranging between .76 and 1. Administration order, age, gender, and Internet skills did not extensively modify the results [36].

Response consistency between data from the personal Social Security number and information collected through the sociodemographic questionnaire was estimated among 84,442 subjects (84.34%, 84,442/100,118). A total of 5141 subjects (6.09%, 5141/84,442) had at least one discordant data value. Sex, age, education, and employment were associated with the response consistency patterns [35].

#### Dietary Data

The comparison of self-reported dietary data against urinary biomarkers (Table 2) revealed that, on an average, men underreported protein intake, whereas women underreported both protein and sodium intake [23].

**Table 2:** Dietary intake and urinary excretion of protein, potassium, and sodium, NutriNet-Santé study, 2013^a^.

Nutrients and indicators		Men (n=102)			Women (n=91)		
		Mean	Lower value of the 95% CI	Upper value of the 95% CI	Mean	Lower value of the 95% CI	Upper value of the 95% CI
**Protein**							
	Mean 24-h U^b,d^ (g/day)	101.7	62.3	166.2	77.4	45.8	130.5
	Mean 24 h^b,e^ (g/day)	88.6	83.9	93.7	68.8	65.1	72.8
	Difference %^c^	−14.4	−18.2	−10.3	−13.9	−18.3	−9.3
**Potassium**							
	Mean 24-h U^b^ (mg/day)	3357	3189	3535	2843	2685	3010
	Mean 24 h^b^ (mg/day)	3444	3279	3618	2739	2607	2879
	Difference %^c^	2.6	−1.7	7.1	−3.6	−8.9	1.9
**Sodium**							
	Mean 24-h U^b^ (mg/day)	3578	3320	3856	2996	2790	3217
	Mean 24 h^b^ (mg/day)	3503	3271	3752	2747	2567	2941
	Difference %^c^	−2.1	−9.2	5.6	−8.3	−15.7	−0.2

^a^Dietary intake values are mean values across 3 24 h, and urinary excretion is the mean of 2 24-h urine samples.

^b^Geometric means based on log-transformed data.

^c^Mean difference in percentage calculated from the log-ratio of mean reported intake (24 h) over mean biomarker intake (24-h Us).

^d^24-h U, 24-h urine collection.

^e^24-h, 24-h dietary record.

Relative differences between reported and “measured” intakes were −14.4% (protein), 2.6% (potassium), −2.1% (sodium) for men, and −13.9%, −3.6%, and −8.3% for women. Misreporting was not associated with body weight status.

Among men, the investigation revealed Spearman correlations ranging between .20 (for vegetables and plasma vitamin C) and .55 (for fish and plasma docosahexaenoic acid [DHA]). Among women, these correlations were generally lower and ranged from .13 (nonsignificant; for vegetables and plasma vitamin C) to .54 (for fish and eicosapentaenoic acid [EPA]+DHA). Regarding micronutrients, adjusted correlations of self-reported intakes with plasma biomarkers ranged from .36 (EPA) to .58 (vitamin C) in men and from .32 (vitamin C) to .38 (EPA) in women [24].

Next, the agreement between the 24-h Web tool data and data obtained via telephone interviews was very high [37], with ICCs ranging from .5 for fats or sauces (among both genders), breakfast cereals, cakes or biscuits or pastries and dairy (women only to .9 for fruits, pulses (among both genders), breakfast cereals, alcoholic drinks, and meat (among men). For nutrient intake, energy-adjusted Pearson correlation coefficients ranged from .6 for polyunsaturated fatty acid, retinol, vitamin E, and sodium (among women) to .9 for a large number of different nutrients. Note that women participating in this comparative study reported higher intake of cakes, biscuits, or pastries when using the Web interface than during the telephone interview with a dietician, indicating that social desirability bias may have been lower in the Web-based version as compared with the traditional version. The acceptability of the Web-based nutrition assessment tool was high, with 92.74% (115/124) of participants judging the Web interface as user-friendly and 66.13% (82/124) preferring the Web-based method over the interview.

The estimation of the financial cost related to the implementation and use of the Web-based dietary questionnaire compared with the cost associated with the standard assessment involving dieticians (for 1 24-h dietary record/recall) are presented in Table 3.

**Table 3:** Cost estimation for traditional and Web-based assessment^a^.

Questionnaire		Cost
**Traditional methods for baseline data assessment**		
	Dietary data, interview (for 1 24-h recall) comprising the salary of the dietitians, telephone expenses, and cost of printing and sending the picture booklet	€38.1/subject
	Anthropometric data, paper version comprising printing, postage for sending and returning the questionnaire, and double data entry	€9.9/subject
	Sociodemographic data, paper version comprising printing, postage for sending and returning the questionnaire, and double data entry	€16.5/subject
	Total for 100,000 subjects	€6,450,000
**Web-based method**		
	NutriNet-Santé Web-based platform comprising the whole study process: secure registration system, development, and administration of baseline questionnaires (including three 24-h records), license, equipment, and hosting) No supplementary cost for an additional dietary assessment	€380,000

^a^Financial estimation using cost in 2009.

**Table 4:** Advantages of Web-based cohort studies for data collection with respect to traditional modes of epidemiological research.

Problematic aspects encountered in epidemiological studies	Advantages of Web-based cohort studies
Representativeness	The use of Internet as the exclusive mode of follow-up was a decisive reason for participation, in particular for men, young individuals, and obese persons Television broadcasts may help to further increase the proportion of population groups that are less likely to participate in cohorts of volunteers (young people, elderly, men, and low socioeconomic status groups) Individuals with a lower computer literacy level might participate Individuals with hearing and other disabilities might participate
Data collection	Reduced logistic, personnel, material, and financial burden of large epidemiological studies Reduction of data entry errors as compared with paper questionnaires
Acceptability	Data entry can be rendered easier and more pleasant by the use of well-designed interactive interfaces and videos, unlike paper questionnaires
Data management	Data treatment can be directly incorporated into the software, leading to rapid availability of the collected information
Validity of nutritional and anthropometric data	High or similar quality as in conventional studies Concerning nutritional data, social desirability bias may be lower in Web-based studies (in particular among women)
Development of new assessment tools	Possibility to rapidly test and implement new assessment tools, protocols, and so on.
Health events assessments	Possibility to rapidly match participant data with different medical registries

#### Anthropometric Data

The validation study showed high ICCs, ranging from .94 for height to .99 for weight, and the concordance for BMI classification was 93% (sensitivity 88% and specificity 99%). However, we observed a slight underreporting of weight and overreporting of height, leading to an underreporting of BMI, which was more pronounced among obese participants. Web-based and face-to-face self-reports of weight and height were almost perfectly concordant (classification agreement was 98.5%).

In the comparative study, agreement between the Web-based version of the self-administered anthropometric questionnaire and the paper-based version of the same questionnaire was very high, with ICCs ranging from .86 to 1 and kappa statistics ranging from .69 to 1 for continuous and categorical variables, respectively [27].

Overall, based on the results of the methodological studies carried out in the NutriNet-Santé study, we provide new insights with respect to Internet use in epidemiological research (Table 4).

## Discussion

### Principal Findings

Overall, the presented findings support an acceptable to high quality of data collected in a large and heterogeneous Web-based cohort with a substantially reduced financial burden. Concordance assessed in objective validation studies and in method comparison studies was high. Moreover, the presented results indicate that Web-based studies may help reduce social desirability biases.

It appears that the validity of the collected data was comparable with that in conventional research (based on interviews or paper-and-pencil tools), or even higher. For instance, acceptable to moderate validity of nutrient intake estimation was observed. Although the observed correlation coefficients may be considered as relatively low in absolute terms, previous validation studies have reported correlations of a similar magnitude [38-41]. Next, bias associated with social desirability, inherent in studies based on interviews, is likely lower in Web-based studies due to higher perceived anonymity particularly among women [6].

Cohorts of volunteers like the NutriNet-Santé study tend to have a distribution of sociodemographic profiles that diverges from the general population [1]. In particular, prospective nutritional cohorts relying on volunteer-based samples have been criticized with respect to their strong susceptibility to self-selection bias. It has been shown that women, older individuals, and married individuals were more prone to enroll in epidemiological studies, irrespective of the research topic, whereas at-risk populations were less likely to participate [1,42,43]. In addition, it has been postulated that Web-based cohorts including motivated Internet-skilled volunteers may be particularly at risk for such bias as Internet accessibility and patterns of Internet use may vary according to sociodemographic profile [7]. However, in Europe, this additional source of selection bias is likely minor given the wide access to the Internet in all subgroups of the population including people above 55 years (42% in 2011) and those with low education (45% in 2011) [44]. Yet, it is possible that difficulties with Internet use may have caused a number of participants with low Internet skills or Internet access problems, in particular older people, to drop out of the study. Beyond self-reported computer skills, the type of activity (unexpected clicks, nonobservance of instructions, etc) across subgroups also merits evaluation.

Our findings also suggest that the exclusive use of Internet for data collection and follow-up—which implies a largely reduced effort for participants [3,45] and research staff—may help increase the relative proportion of population subgroups (young people, elderly, men) that are usually underrepresented [1,42,43]. Nevertheless, the Web-based design appeared to be a less important motive for participation among individuals in “low” compared with “high” occupational categories. Besides, the use of a wide range of recruitment channels beyond the Internet may help further diversify exposure profiles. Television, which is a wide-reaching medium in France, may be a particularly useful instrument for improving the recruitment of population subgroups that tend to be underrepresented in volunteer-based epidemiologic studies such as low educated individuals or unemployed individuals.

### Advantages and Limitations

Web-based cohorts have the potential to include a large number of participants, including the hard-to-reach subgroups. Yet, as those who are willing to participate in a Web-based cohort study probably differ from the general population, other means to specifically increase opportunities for participation in epidemiological Web-based studies among low socioeconomic groups is needed. In that context, statistical weighting methods are an important and efficient approach to help counterbalance limitations concerning the external validity of descriptive epidemiological studies.

A key advantage of Web-based research is related to data entry. By comparing the Web-based version of baseline questionnaires with the traditional paper-and-pencil version used in methodological studies [27,36], we also showed that a substantial number of data entry mistakes, missing values, and inconsistent or abnormal values were found in the paper version, whereas they were nonexistent in the Web-based version due to integrated controls. Computerized data entry also obviates the need for data coding or entry personnel. This provides major advantages concerning the logistic, personnel, material, and financial burden of large epidemiologic studies. In addition, a large proportion of data quality control can directly be implemented into the Web-based software that may substantially improve the quality of the collected data [6]. In contrast to a paper questionnaire, Web-based tools can directly alert the participant if unrealistic values are entered or prevent the participant from submitting an incomplete questionnaire. Besides, long questionnaires can be simplified by implementing conditional jumps.

In addition, data entry can be rendered more pleasant by the use of a well-designed interactive interface, audio and visual feedback, and pop-up windows providing additional information regarding potentially complicated elements of the questionnaires. Indeed, our investigation of the quality of the nutritional data collected in NutriNet-Santé showed that a very high percentage of participants perceived the Web-based nutrition assessment tool as user-friendly. A further advantage pertains to the high flexibility of the Web-based platform, allowing rapid implementation of new questionnaires or ancillary protocols. For instance, several questionnaires have been developed or translated in the context of the NutriNet-Santé study, as such a platform permits rapid testing and fine-tuning for the validation of new questionnaires [46,47].

### Future Research

This paper provides an overview of methodological advances in e-epidemiology and fills gaps in knowledge concerning specific methodological aspects of e-cohorts and their design, such as participant selection processes, sample representativeness, diversity of sociodemographic profiles, and the validation of Web-based tools. The development of ad hoc validation and comparative studies may help improve innovative digital tools and to reinforce confidence in data collected using new technologies. This may guide further development and implementation of future e-cohorts and validation studies that are currently scant.

Future directions may include evaluating the consistency of health data collected via different sources (self-reported data, disease registries, or medical databases). It would also be useful to implement ad hoc studies to compare characteristics of hard-to-reach subgroups with those of nonparticipants.

## Abbreviations

BMI: body mass index
DHA: docosahexaenoic acid
EPA: eicosapentaenoic acid
ICCs: intraclass correlation coefficients
