# Metadata Correction of: Lessons Learned From Methodological Validation Research in E-Epidemiology

**DOI:** 10.2196/publichealth.6876

**Published:** 2016-10-31

**Authors:** Emmanuelle Kesse-Guyot, Karen Assmann, Valentina Andreeva, Katia Castetbon, Caroline Méjean, Mathilde Touvier, Benoît Salanave, Valérie Deschamps, Sandrine Péneau, Léopold Fezeu, Chantal Julia, Benjamin Allès, Pilar Galan, Serge Hercberg

**Affiliations:** ^1^Équipe de Recherche en Epidémiologie Nutritionnelle (EREN), Centre de Recherche en Epidémiologie et Statistiques, COMUE Sorbonne Paris Cité, Inserm (U1153), Inra (U1125), CnamUniversité Paris 13BobignyFrance; ^2^Ecole de Santé Publique, Centre de Recherche en Epidémiologie, Biostatistiques et Recherche CliniqueUniversité Libre de BruxellesBruxellesBelgium; ^3^Unité de Surveillance et d’Epidémiologie Nutritionnelle (USEN), Institut de Veille Sanitaire, Centre de Recherche en Epidémiologie et Statistiques, COMUE Sorbonne Paris CitéUniversité Paris 13BobignyFrance; ^4^Hôpital AvicenneDépartement de Santé PubliqueBobignyFrance

The authors of “Lessons Learned From Methodological Validation Research in E-Epidemiology” (JMIR Public Health Surveill 2016;2(2):e160) made a mistake in the name of author Katia Castetbon. The author's name should be Katia Castetbon instead of Katia Castebon.

This correction has been made in the online version of the paper on the JMIR Public Health and Surveillance website on October 31, 2016 together with publishing this correction notice. Because these were made after submission to PubMed and other full-text repositories, the correction notice has been submitted to PubMed, and the original paper has been resubmitted to PubMed Central. The corrected metadata have also been resubmitted to CrossRef.

